# Case Report: Spesolimab for pyoderma gangrenosum in an undifferentiated oligoarthritis patient receiving anti-IL-17 therapy

**DOI:** 10.3389/fimmu.2025.1581996

**Published:** 2025-04-07

**Authors:** Weiwei Xin, Liyang Gu, Fang Du, Ting Li, Shuang Ye

**Affiliations:** ^1^ Department of Orthopedics, Renji Hospital, School of Medicine, Shanghai JiaoTong University, Shanghai, China; ^2^ Department of Rheumatology, Renji Hospital, School of Medicine, Shanghai JiaoTong University, Shanghai, China

**Keywords:** Spesolimab, anit-IL36R, pyoderma gangrenosum, IL-17, undifferentiated oligoarthritis, Ixekizumab, case report

## Abstract

Pyoderma gangrenosum (PG) is a classic neutrophilic dermatosis often associated with inflammatory conditions, frequently leading to misdiagnosis and delayed treatment. Drug-induced and postoperative are two potential triggers of PG. A 70-year-old woman, who had been treated with Ixekizumab for undifferentiated oligoarthritis, presented with cribriform violaceous ulcers on her right posterior ankle after 8 months of debridement. A skin biopsy revealed a predominant neutrophilic infiltrate with no signs of infection after extensive microbiology investigation. The patient was diagnosed with pyoderma gangrenosum (PG) and achieved remission after receiving three doses of 900 mg intravenous Spesolimab every 4 weeks, along with discontinuation of Ixekizumab. The case highlights the successful use of Spesolimab (anti-IL36R) in treating PG and explores the potential “paradoxical phenomenon” linked to anti-IL-17 therapy, providing novel insights into immune dysregulation and therapeutic strategies.

## Introduction

Pyoderma gangrenosum(PG) is a typical neutrophilic dermatosi, frequently associated with inflammatory disorders (1). Although its pathophysiology remains incompletely understood, multiple proinflammatory cytocines, such as Tumor necrosis factor-α (TNF-α), Interleukin-8 (IL-8), IL-17, IL-23, and IL-36, and different predisposing genetic background has been implicated in the disease process (2, 3). Given its diverse clinical manifestations and lack of characteristic laboratory findings, PG is often misdiagnosed and experiences delayed diagnosis. Current treatment options for PG are limited and often involve systemic corticosteroids, immunosuppressants, and biologic agents targeting specific inflammatory pathways. However, these therapies are not universally effective, highlighting a significant unmet need for targeted treatments. IL-36 has been demonstrated to play a critical role in neutrophil-mediated inflammation. Spesolimab, an anti-interleukin-36 receptor(IL-36R) monoclonal antibody, received FDA approval for generalized pustular psoriasis in 2022 (4). Here, we present the challenging clinical course of a patient who developed pyoderma gangrenosum during anti-IL-17 therapy for undifferentiated oligoarthritis, culminating in complete resolution following targeted IL-36 pathway inhibition with spesolimab.

## Case description

**Figure 1:**
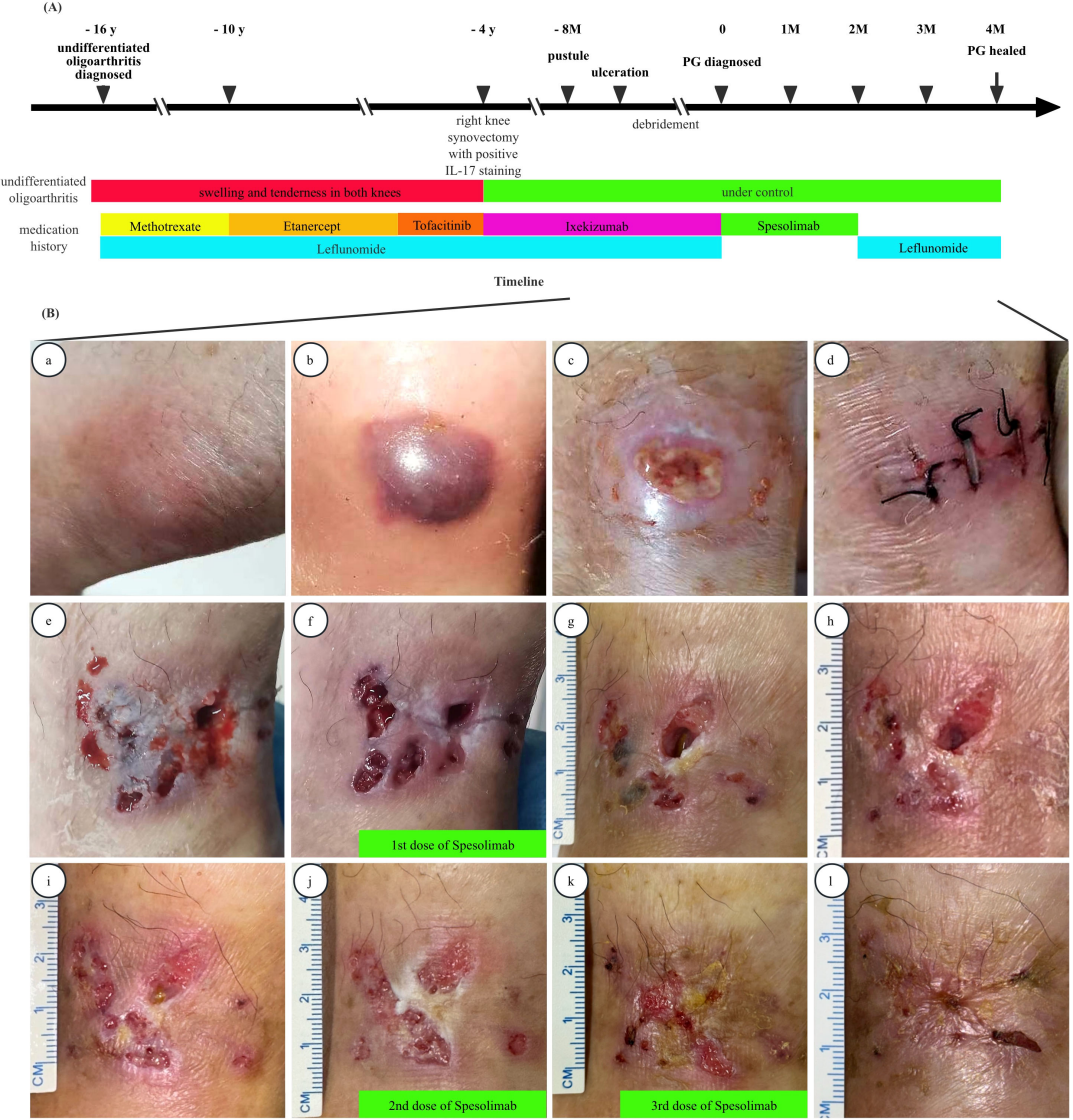
**(A)** Timeline of the disease. **(B)** The evolution of pyoderma gangrenosum: (a-c) Progression from pustules to ulcer within weeks on the right posterior ankle. (d-f) The undermined ulcer expanded after debridement. (g-i) the size of the lesion significantly reduced within 4weeks of Spesolimab infusion. (j-l) The lesion gradually healed and re-epithelialized in the following 3months. PG, pyoderma gangrenosum.

A 70-year-old woman presented to the rheumatology clinic with an 8-month history of refractory ulcers on her right posterior ankle. Sixteen years prior to this presentation, she was diagnosed as undifferentiated oligoarthritis based on swelling and tenderness in both knees, with negative rheumatoid factor and anti-cyclic citrullinated peptide antibody (ACPA). No psoriasis or inflammatory bowel disease(IBD) or low back pain were reported along with a negative human leukocyte antigen (HLA-B27). The patient had an inadequate response to conventional disease-modifying anti-rheumatic drugs (DMARDs), such as Methotrexate and Leflunomide, and was subsequently switched to a tumor necrosis factor (TNF) inhibitor (Etanercept) with Leflunomide, and then a Janus kinase inhibitor (JAK) inhibitor (Tofacitinib) with Leflunomide. However, these treatments yielded suboptimal results, with no significant improvement in knee symptoms, although no adverse effects were reported. Four years prior to the current presentation, due to worsening swelling and pain in the right knee joint that impaired her daily living activities, she underwent a synovectomy. Given the lack of response to TNF and JAK inhibitors, IL-17 staining was performed on the synovial tissue, which yielded a positive result. Consequently, she was started on Ixekizumab 80mg/month in combination with Leflunomide 20mg/d (**Figure 1A**). Her disease was gradually under control and her daily activity was returned to normal, e.g., go hiking, with a significantly improved quality of life.

Unfortunately, 8 months prior to the current presentation, without trauma or tick-bite, a tender pustule appeared on her right posterior ankle, which progressed into a violaceous blister, and then an ulcerative lesion (**Figures 1A, B** a-c). After multiple debridements, the undermined ulcer expanded. The non-healing deep ulcerative lesion with surrounding shallow cribriform violaceous ulcers can be appreciated in the subsequent 7 months (**Figures 1A, 1B** d-f). There was no relevant family history.

## Diagnostic assessment

During hospitalization, laboratory tests revealed a slightly elevated erythrocyte sedimentation rate (ESR) at 25 mm/h, with no other hematological abnormalities. Extensive microbiological investigations ruled out infection. Histopathological examination of a skin-biopsy obtained from the border of the lesion revealed localized exudation and necrosis, dermal fibroblast hyperplasia with collagenization, and a predominantly neutrophilic infiltrate with scattered lymphocytes and eosinophils, as well as occasional multinucleated giant cells. Periodic acid-Schiff diastase (D-PAS) and Alcian blue stain (AB) stains were negative. Direct immunofluorescence was also negative for complement 3 (C3), immunoglobulin (Ig) G, IgM, IgA and fibrinogen.

Given the patient’s progressive clinical course, absence of infections or malignancy, reddish-violaceous wound border, tender and undermined ulcer, suppurative inflammation on histopathology, and background of undifferentiated oligoarthritis, her PARACELSUS score (5) were 14, supporting a diagnosis of pyoderma gangrenosum (PG).

After written informed consent obtained, the patient discontinued Ixekizumab and Leflunomide, and was started on intravenous Spesolimab for 3 doses of 900 mg every 4 weeks, albeit IL-36RN mutation sequencing was turned out negative. Within 4 weeks of the first Spesolimab infusion, the lesion size significantly decreased (**Figures 1A, B** g-i). Over the following 3 months, the ulcers gradually healed with re-epithelialization (**Figures 1A, B** j-l). Following the final dose of spesolimab, Leflunomide was reintroduced for undifferentiated oligoarthritis.

## Discussion

PG is a rare, inflammatory neutrophilic dermatosis characterized by recurrent skin ulcers with a chronic course. It has a reported prevalence of 5.8 per 100,000 individuals and an annual incidence of 3-10 cases per million (1). PG is frequently associated with systemic inflammatory disorders, such as rheumatoid arthritis(RA) and IBD. In a restricted subset of cases, PG represents a component of distinct autoinflammatory syndromes, including PAPA(pyogenic arthritis, pyoderma gangrenosum, and acne), PASH(pyoderma gangrenosum, acne, and suppurative hidradenitis),and PAPASH(pyoderma gangrenosum, acne, pyogenic arthritis, and suppurative hidradenitis) (6).

The pathogenesis of PG remains incompletely understood, but dysregulation of pro-inflammatory cytokines, including TNF-α, IL-1, IL-17, IL-23, and IL-36, has been implicated in the disease process. Genetic predisposition and dysregulation of both innate and adaptive immune responses also play a significant role. Variant enrichment analysis has identified disruptions in multiple molecular pathways, including immune regulation, cell metabolism, structural function, cell signaling, and other pathways in PG (3). Additionally, keratin variants, such as KRT18 rs77999286, have been linked to multilesional disease, suggesting a potential genetic component in disease severity and progression (2).

Clinically, PG typically presents on the lower limbs or trunk, but can occur at any site (1). Lesions often begin as sterile small pustules, papules, or bloody blisters, which rapidly progress to painful, enlarging ulcers. These ulcers are characterized by a reddish purple, elevated, and undermined margin. In addition to the classic ulcerative form, bullous, pustular, vegetative, peristomal and postoperative variants have also been described (6). Although bacterial colonization is common secondary to ulceration, PG is classified as a non-infectious neutrophilic dermatosis. The term “pyoderma” historically reflected the misconception of bacterial etiology, but current understanding places PG within the spectrum of autoinflammatory skin diseases. Histologically, PG exhibits biphasic changes: (1) central necrotizing suppurative inflammation with collagenolysis, hemorrhage, and neutrophil infiltration, and (2) peripheral Sweet syndrome-like vascular reactions with neutrophilic margination. Direct immunofluorescence often reveals IgM, C3, and fibrin deposition in vessel walls, while IgG or IgA deposition is rare (7). Although these findings are not pathognomonic, they are valuable for excluding other conditions, such as malignancies, autoimmune blistering diseases, lupus erythematosus, and vasculitis.

Diagnostic criteria for PG remain debated, but the PARACELSUS score is considered highly effective and sensitive (2). It incorporates factors such as progressing disease, assessment of relevant differential diagnoses, a reddish-violaceous wound border, amelioration, characteristically irregular ulcer shape, extreme pain, localization of lesion at site of trauma, suppurative inflammation on histopathology, undermined wound border, and systemic disease associated to those clinically diagnosed with PG.

In this case, the patient presented with rapidly progressive ulceration following a tender pustule on the right posterior ankle. No acne, comedones, or family history of similar conditions were observed (6). Histopathological examination revealed dense neutrophilic infiltrates without evidence of vasculitis, and microbial studies were negative. These clinical and histological features were consistent with a diagnosis of PG, according to the PARACELSUS score. The rapid and complete resolution of the ulcers following Spesolimab therapy further supported the diagnosis.

Two potential triggers were considered in this case: drug-induced PG and postoperative PG. Drug-induced PG has been associated with agents such as tumor necrosis factor (TNF) inhibitors, molecular-weight compounds, and Ipilimumab (8–11). Notably, IL-17 inhibitors, including Ixelizumab, have been linked to paradoxical inflammatory reactions, including new-onset or exacerbated IBD and psoriasis (12). Aromolo et al. reported a case of PG, who was treated with Brodalumab for psoriasis (13). In this case, the patient’s use of Ixekizumab for undifferentiated oligoarthritis may have contributed to PG development by disrupting the IL-23/IL-17 axis, leading to IL-23 upregulation and subsequent neutrophilic inflammation (13). Additionally, postoperative PG exacerbation has been reported, likely due to tissue manipulation, inflammation, and surgical stress (14, 15). The greater the amount of tissue manipulation, which is thought to involve dysfunction and upregulation of neutrophil activities, the higher the likelihood of subsequent PG. In this case, multiple debridements may have exacerbated the condition.

The management of PG typically involves systemic corticosteroids and immunosuppressants. For mild or early disease, topical therapies may suffice, while moderate to severe or progressive cases often require systemic agents, including biologics. Neutrophil-mediated inflammation is central to PG pathogenesis. IL-36, a pro-inflammatory cytokine in the IL-1 family, plays a key role by amplifying inflammation through neutrophil recruitment and activation. IL-36 induces the expression of cytokines such as IL-8, TNF, CXCL-8, IL-17A, and IL-6, while also promoting Th1 differentiation and inhibiting Treg differentiation, thereby exacerbating inflammation and disease progression.

Spesolimab, an IL-36 receptor antagonist approved for generalized pustular psoriasis, has shown promise in the treatment of PG, as evidenced by several case reports and our patient’s response (16, 17). Ongoing clinical trials (NCT06624670, NCT06092216) aim to further elucidate the role of Spesolimab in PG treatment, providing hope for more targeted and effective therapies.

Increasing awareness of PG among non-dermatologists is critical for early recognition, accurate diagnosis, and targeted treatment to achieve clinical remission. While Spesolimab shows promise, its efficacy in PG requires further validation through additional clinical data. Physicians should remain vigilant for paradoxical inflammatory reactions, such as PG, in patients receiving anti-IL-17 therapies for arthritis or other conditions. Continued research into the underlying mechanisms and optimal treatment strategies for PG is essential to improve patient outcomes.

## Data Availability

The raw data supporting the conclusions of this article will be made available by the authors, without undue reservation.
